# Parents' Distress and Demands for Children With Cancer‐Related Cognitive Impairment in Western China: A Qualitative Study

**DOI:** 10.1002/cam4.70257

**Published:** 2024-10-10

**Authors:** Tao Liu, LiFang Xu, Yuqing Shen, Zefang Chen, Juan Yao, Lin Mo

**Affiliations:** ^1^ School of Nursing Chongqing Medical University Chongqing China; ^2^ Out‐Patient Department Children's Hospital of Chongqing Medical University Chongqing China; ^3^ School of Nursing Children's Hospital of Chongqing Medical University Chongqing China; ^4^ Hemato‐Oncology Department Children's Hospital of Chongqing Medical University Chongqing China

**Keywords:** cancer, children, cognitive impairment, parents, qualitative study

## Abstract

**Backgroud:**

Cancer‐related cognitive impairment is one of the common complications in children with cancer, but our understanding of their experience with cognitive deficits remains limited. From the perspective of parents, this study aimed to explore the distress and demands faced by children with cancer‐related cognitive impairment, to provide references for developing targeted intervention strategies for cancer children.

**Methods:**

We used a purposeful sampling method to conduct semi‐structured interviews with the parents of 18 children with cancer‐related cognitive impairment. The transcripts were analyzed using Colaizzi's method.

**Results:**

Three categories and 11 subcategories were obtained from the data analysis, including diverse levels of cognitive impairment in children (speech communication difficulties, impaired executive function, attention deficit, and intellectual disability), persistent negative emotions (anxiety and worry, complaints and resentment, negative avoidance and positive experiences after psychological adjustment), multiple needs (need for disease information, need for professional management, and an urgent need for more external support).

**Conclusion:**

Parents of children with cancer‐related cognitive impairment face a significant psychological burden, coupled with confusion and numerous inquiries regarding the symptoms and management of their children's cognitive impairment. It is imperative for medical professionals to strengthen the dissemination of information related to cancer‐associated cognitive impairments, while promptly recognizing and intervening in related symptoms. Rational allocation of resources, establishment of targeted support systems, and enhancement of social acceptance may be the key points that policymakers could consider. These efforts hold immense significance, as they play a pivotal role in elevating cognitive capabilities and enhancing the overall quality of life for children with cancer.

## 1 Introduction

Cancer is one of the leading causes of death among children worldwide. It is revealed that approximately 160,000 children are diagnosed with cancer annually, marking an upward trend year on year [1, 2]. Although advancements in treatment have led to a 5‐year survival rate exceeding 80% for childhood cancer [3], approximately two‐thirds of survivors still experience long‐term complications due to their treatment. Among these, cancer‐related cognitive impairment stands out as the most common and severe neurological complication [4, 5]. The occurrence rate of cancer‐related cognitive impairment among survivors of children with cancer ranges from approximately 40%–60%, particularly prevalent in cases of brain tumors and leukemia [6]. It is primarily manifested as altered cognitive functions, including decreased abilities in neurocognitive domains such as attention, executive function, and learning capacity [7, 8]. Some previous studies have suggested that as these children with cognitive impairments age, they face an increased risk of lower educational attainment, professional achievements, and employment rates. Additionally, they are at a greater risk of experiencing discrimination in their future work and life compared to their peers, ultimately leading to unfavorable outcomes such as inability to live independently, difficulties integrating into society, and decreased quality of life [9, 10, 11].

A profound analysis of the symptomatic experiences and needs of children with cancer‐related cognitive impairments is pivotal in guiding the implementation of future intervention [12, 13, 14]. Nevertheless, the majority of current research on pediatric cancer‐related cognitive impairments predominantly used quantitative methodologies to investigate its influencing factors and prognosis [15, 16], thereby failing to comprehensively reflect the symptomatic burden and needs faced by patients and their families. Qualitative research offers a more detailed understanding of the life experiences of children with special needs, uncovering previously unrecognized feelings and demands [17]. Furthermore, a previous study has shown that it is necessary to manage cancer‐related symptoms by integrating the perspectives of children, parents, and healthcare professionals [18]. Although a limited number of scholars have described the life experiences and needs of children with cancer‐related cognitive impairments from the perspectives of cancer survivors and healthcare professionals [19, 20, 21], these endeavors have nonetheless laid a foundation for this present study to comprehend the cognitive impairment experiences and needs of children with cancer specifically from the perspective of parents.

Therefore, this study used a qualitative research approach to conduct semi‐structured interviews with the parents of children with cancer‐related cognitive impairments, aiming to gain an understanding of the symptomatic experiences of these children from the perspective of their parents and to delve into the challenges and needs encountered during the caregiving process, so as to provide valuable insights for the allocation of rehabilitation resources and other support policies aimed at improving the lives of childhood cancer survivors.

## 2 Materials and Method

### 2.1 Design

This study used a qualitative exploratory research design involving 18 parents to explore the distress and demands experienced by parents of children with cancer‐related cognitive impairment. Qualitative research offers a multidimensional and in‐depth exploration and analysis of the phenomenon under study, enabling participants to share their perspectives and real‐world experiences [22].

### 2.2 Participants

The parents in the study lived with their children in western China, including four provinces: Chongqing, Sichuan, Yunnan, and Guizhou. The participants were selected from a children's hospital in Chongqing, which is the largest pediatric cancer center in Western China. Drawing upon rigorously defined inclusion and exclusion criteria, the parents of pediatric cancer patients hospitalized in this hospital between August and October 2023 were meticulously chosen to take part in this study. The process of cognitive development among children is segmented into distinct age brackets, with the age of 8 serving as a pivotal milestone. First, children who have surpassed the age of 8 exhibit a brain development level that is comparable to that of adults [23]. Furthermore, previous research has demonstrated that these older children possess the capability to independently articulate and report their psychological sentiments [24]. For these reasons, the inclusion criteria for children with cancer were as follows: (1) Duration of cancer treatment > 6 months; (2) age between 3 and 8 years old; (3) selecting the appropriate scale based on children's Age. Children aged 3–6 undergo assessment with the Wechsler Preschool and Primary Scale of Intelligence—Fourth Edition (WPPSI‐IV), while children aged 6–8 are evaluated using the Wechsler Intelligence Scale for Children—Fourth Edition (WISC‐IV). Based on the recommended standards of the international cognitive and cancer collaborative groups [25]. The participants scored > 70 on two or more subtests of the Wechsler Intelligence Scale, fourth edition; (4) the respondent being a direct relative of the patient and cohabiting for over a year. Exclusion criteria were as follows: (1) the presence of other diseases affecting cognitive function, such as attention deficit hyperactivity disorder, autism, and cerebral palsy; (2) parents with major illnesses (such as advanced cancer); (3) Due to personal reasons, caregiving was replaced or interrupted midway.

### 2.3 Data Collection

In accordance with the research objectives, we conducted a comprehensive review of pertinent literature. Through rigorous discussions among the research team members, coupled with the valuable insights of two nurses possessing over 10 years of clinical experience and profound expertise, we initially formulated a guide for interview questions. To further guarantee the precision and applicability of these questions, we deliberately chose two parents of children with cancer to participate in a preliminary interview. Following deliberations and adjustments by the research team, the definitive interview question guide was established (Table 1). During the interview process, all participants were asked the relevant questions from the guide, but the order of the questions was flexibly adjusted based on the actual situation to ensure the smooth progress of the interview.

**TABLE 1:** Semistructured interview guide.

Interview outline
1. Have you noticed a decline in your child's attention span, executive function, or attention span in daily learning and life? Please explain. 2. Do the above symptoms affect the daily life of the child and family? Please describe it in detail? 3. What do you think are the causes of the above symptoms? 4. What worries you most about these symptoms in your child? Please explain. 5. What strategies did you employ to tackle the issue, and did you explore any external support or resources? 6. What specific information and support are you most eager to acquire? Please explain.

After obtaining ethical approval from the Hospital, semistructured face‐to‐face interviews were conducted to collect data [26]. The researcher (the first author of this article) introduced himself and briefly explained the purpose and process of the study. The duration of each interview ranged from 40 to 60 min. All interviews were recorded with the consent of the participants. During the interviews, the researcher used notes to record the participants' behaviors, attitudes, and psychological states, as well as any information that could facilitate the analysis of the interviews. Given the sensitivity of the topic, most interviews were conducted in the evening in quiet meeting rooms. During the interviews, no one else was present besides the researcher and the participant, which facilitated the participants' sharing of their experiences.

The researchers were residents of the surveyed area and were fully familiar with the customs and culture of the region. They recruited eligible participants during their clinical work. Data saturation was achieved after conducting interviews with 18 individuals, as the codes derived from these interviews began to repeat, and the researchers confirmed that no new codes were emerging through further interviewing. Subsequently, theoretical saturation occurred, prompting the researcher to halt the interview process since no new information was being obtained by continuing [27]. The interviews were conducted in Chinese and then translated into English by the first author from the beginning to the end, with the translation being validated by the third author.

### 2.4 Data Analysis

The interview data were analyzed by two researchers (the first and second authors), adhering strictly to Colaizzi's seven‐step approach [28]. This approach encompassed the following steps: meticulously perusing all transcribed materials, pinpointing statements of profound significance, coding recurrent themes, clustering the codes to form cohesive themes, offering detailed descriptions of the thematic content, discerning similar perspectives to refine the themes, and validating the findings with the participants. Data management was executed utilizing the qualitative research analysis software NVivo 12.0. Two raters sorted and analyzed the data following the aforementioned steps. Upon achieving high consistency in their results, the reliability of the qualitative research was deemed ideal, and the outcomes were adopted to ensure the validity of the approach. After analysis, the researchers verified the final results with both the raters and interviewees, ensuring that they were consistent with the participants' feelings and guaranteeing the authenticity and accuracy of the data. During information summarization, all authors engaged in repeated discussions until a consensus was reached.

### 2.5 Credibility

This study adhered to the 32 items outlined in qualitative research reporting guidelines [29]. Given the qualitative research conducted by our team in the field of childhood cancer and the involvement of these parents in the clinical treatment and care of their children, the researchers were able to collect information from these parents, as the research subjects.

To guarantee the authenticity and credibility of the emergent themes, the data were encoded and analyzed, incorporating the insights of qualified qualitative researchers, medical practitioners who have worked and researched extensively in the psychological realm of childhood cancer, and all contributing authors. This holistic approach ensured examination and classification of the themes, leading to a comprehensive understanding of the data.

### 2.6 Ethical Considerations

This study was approved by the hospital's ethics committee (approval number: 2023.288‐1). People who expressed interest in the study were informed of the information about this study and the contact information of the data protection officer, and participants' privacy, confidentiality, and anonymity were guaranteed. They were also informed that their voluntary participation would not be condemned. Those who agreed to participate signed a written informed consent form.

## 3 Results

### 3.1 Characteristics of Parents and Children With Cancer

Eighteen parents of children with cancer participated in the interview. The general information of children with cancer and their parents is shown in Table 2.

**TABLE 2:** Characteristics of parents and children with cancer.

Sociodemographic data	Frequency (*n*, %)
Parent' age	
< 30	6 (33.33)
31–40	8 (44.44)
> 40	4 (22.22)
Parent gender	
Male	5 (27.78)
Female	13 (72.22)
Parent education level	
Illiteracy	2 (11.11)
Primary	5 (27.78)
Secondary	6 (33.33)
Tertiary	5 (27.78)
Employment status	
Employed	2 (11.11)
Business	3 (16.67)
Not employed	7 (38.89)
Subsistence farming	6 (33.33)
Relationship with the child	
Mother	12 (66.67)
Father	6 (33.33)
Child age	
3–6	12 (66.67)
6–8	6 (33.33)
Child gender	
Male	10 (55.56)
Female	8 (44.44)
Type of cancer	
Leukemia	8 (44.44)
Cerebroma	7 (38.89)
Other	3 (16.67)
Duration of cancer treatment	
> 6 months	5 (27.78)
6–12 months	7 (38.89)
> 12 months	6 (33.33)
Wechsler intelligence scale score	
> 70	7 (38.89)
75–80	8 (44.44)
< 80	3 (16.67)

### 3.2 Themes and Subthemes

We have identified three themes to describe the parents' distress and demands regarding the cognitive impairments of children with cancer. Each theme has its own subthemes, as outlined in Table 3.

**TABLE 3:** Themes and subthemes of parents' distress and demands with cognitive impairment in children with cancer.

Theme	Subtheme
1. Diverse levels of cognitive impairment in children	1a: Speech communication barriers 1b: Impaired executive function 1c: Attention deficit 1d: Intellectual disability
2. Persistent negative emotions	2a: Anxiety and worry 2b: Complaints and resentment 2c: Negative avoidance 2d: Positive experiences after psychological adjustment
3: Multiple demands	3a: Needs for disease information 3b: Need for professional management 3c: Urgent need for more external support

#### 3.2.1 Theme 1: Diverse Levels of Cognitive Impairment in Children

*1a: Speech communication barriers*: In this study, a total of 12 parents reported that their children's language expression abilities were poor compared to children of the same age group. Speech disorders such as unclear pronunciation, difficulty in forming words, slow speech, and naming difficulties affected the children's communication in daily life, causing inconvenience for both the children and the parents.

“Sometimes she speaks in a grumbling manner, sometimes she hums… When she gets anxious, she can suddenly utter a few words, but people who do not spend every day with her can barely understand her.”(P3).

“I feel that his communication skills are far behind those of children his age. He seems to have trouble organizing his language… Every time I communicate with him, it is quite challenging. When he gets anxious, I also get anxious.”(P4).

*1b: Impaired Executive Function*: Executive function is a part of higher cognitive functions, including working memory, inhibitory control, reasoning, and problem‐solving. Children with impaired executive function often exhibit behaviors lacking discipline and have difficulties planning and strategizing when doing tasks. Eleven parents in the study reported that their children had symptoms of impaired executive function in daily life.

“Every time I ask him to do something, I have to call him many times before he responds. Sometimes he pretends not to hear me… I feel like he was not like this before he got sick. He used to be a very obedient child.”(P5).

“You cannot ask him to complete more complex tasks, for example, he cannot follow the steps to complete homework.”(P8).

*1c: Attention Deficit*: In the study, eight parents reported noticing problems with concentration in their children compared to their peers.

“He only has a three‐minute attention span for everything… When he was in school, the teacher mentioned that he was often distracted during most of the class.”(P1).

“I feel that his ability to focus is shorter than other children's… He has difficulty concentrating well on any particular task.”(P5).

*1d: Intellectual Disability*: Five parents reported in interviews that their child had an intellectual disability after being diagnosed with cancer.

“I took my child to the pediatric department for an intelligence test after the (cancer) diagnosis, and the doctor told us that the child has a slight intellectual disability.”(P3).

“An intelligence test was conducted during school enrollment, and the results showed that the child's intellectual level is lower than that of other children.”(P11).

#### 3.2.2 Theme 2: Persistent Negative Emotions

*2a: Anxiety and Worry*: For preschool children, the impact of cognitive impairment primarily manifested in their language communication due to their tender years and limited independent living skills. Consequently, parents primarily fretted over the daily welfare of these young children. However, as they grew older and the long‐term consequences of cognitive impairment, parents' anxieties shifted toward the future, specifically wondering whether the condition would hinder the child's academic pursuits, employment opportunities, and their capacity to live independently in adulthood.

“I am quite worried about how this will affect my child's future studies and life. If the symptoms become more severe, will it affect their future development?”(P1).

“This may cause my child to have poor academic performance in the future. What will they do when looking for a job? But I hope it won't be that serious.”(P7).

*2b: Complaints and Resentment*: The treatment of cancer has already taken a toll on the parents, draining them both physically and mentally. The emergence of cognitive impairment symptoms has further added to their burden, leading some parents to feel resentful and full of complaints.

“I thought that once the cancer was treated, it would be fine. Now there is cognitive impairment. It is just unfair.”(P4).

“Why am I so unlucky? Everything bad seems to happen to me. I really feel so frustrated.”(P16).

*2c: Negative Avoidance*: When asked about managing cognitive impairments in children, some parents adopted a passive and avoidant mindset due to lack of confidence in symptom management.

“My child is still so young and has suffered so much from the treatment. I really do not want to add any more burden to his treatment.”(P3).

“As long as my child's leukemia can be cured, it does not matter if he is a little less intelligent in the future.”(P9).

*2d: Positive experiences after psychological adjustment*: In the course of caregiving, parents have experienced positive growth experiences, not only as caregivers but also as individuals. They selflessly dedicated themselves to providing comprehensive care for the child, striving to acquire relevant nursing knowledge to ensure the best possible care. Concurrently, they engage in self‐reflection, delve into their innermost emotions, and embrace the challenges of self‐growth, thereby bolstering their resilience in the face of adversity. This process aligns with the contemporary posttraumatic growth theory in positive psychology.

“There's no other way… We have also learned about remedial measures for cognitive impairment online, and we do some cognitive rehabilitation training for our child at home.”(P8).

“For the sake of our child's future, we will work hard to help them recover.”(P12).

#### 3.2.3 Theme 3: Multiple Demands

*3a: Needs for Disease Information*: During interviews, several parents revealed that they resorted to search engines, WeChat official accounts, and various other platforms to gather knowledge on cognitive decline and training methods for cancer‐affected children. Nevertheless, they conceded that the information and advice dispensed through these mediums often lacked professionalism and credibility, oftentimes inundated with irrelevant or even misleading details. Many parents attributed the emergence of cognitive impairment symptoms solely to the child's nonattendance at school, failing to associate it with cancer. In the present study, 13 parents admitted to being unaware of cancer‐induced cognitive impairment in children, while five parents admitted to encountering occasional online mentions of the condition but disregarding them. Collectively, all parents emphasized the inadequate availability of health‐related information, emphasizing their urgent need for professional medical guidance to satisfy their knowledge requirements.

“Is our child's condition serious? Will it have an impact on the future? How can I remedy it?”(P8).

“What is cognitive impairment? Our child just reacts a little slower. How does that mean cognitive impairment? Please explain this issue to me. Is it serious?”(P11).

The majority of parents held the belief that the resolution of cognitive impairment would follow the cure of cancer. They considered it commonplace for children undergoing cancer treatment to exhibit symptoms such as inattention and compromised executive function. These parents attributed the emergence of these symptoms to the natural course of the disease, assuming that the prolonged hospitalization was the sole culprit behind their child's cognitive decline. They optimistically anticipated that their child's cognitive function would recover upon resuming school, without realizing the urgency of seeking medical attention promptly.

“I feel that these are all caused by chemotherapy, as chemotherapy drugs will inevitably have side effects. But I think these will all improve after chemotherapy is completed.”(P2).

“These are all normal, because the child has been staying in the hospital. Once the illness improves and they return to school, they will be fine.”(P6).

*3b: Need for Professional Management*: The majority of the participants in this study resided in a home care environment, where parents were burdened with a myriad of household chores and caregiving responsibilities alongside managing their child's cancer treatment. Given the widespread lack of knowledge regarding cognitive impairment treatment methods among parents, they actively sought assistance from social services, such as cognitive rehabilitation institutions. However, these institutions often lacked sufficient professionalism in disease care, systematic training, and extensive practical experience. Consequently, they were limited to training children in basic skills like language communication and numerical calculation, with most outcomes being less than satisfactory. Many parents expressed their desire for professional, targeted, and personalized institutions or individuals who could offer guidance, and facilitating cognitive function training for their children while also educating them on cognitive training methods.

“I just feel that if the staff are going to do cognitive training, they should have some basic knowledge. I do not even know if they can train my child in this ability.”(P3).

“I really need professionals to guide us, such as telling us about cutting‐edge research results and how to achieve the best results.”(P17).

*3c: Need for Multifaceted Support*: Children are at a critical stage of rapid development of the nervous system and formation of cognitive function and are more likely to have cognitive impairment after diagnosis of cancer [30, 31]. The immense psychological and economic pressure experienced by parents prompts them to eagerly seek support from schools, society, and other entities. However, a significant proportion of individuals lack a precise and comprehensive understanding of these children, erroneously believing that cancer is incurable and that affected children will be impaired in areas such as intelligence and language communication, leading them to be perceived as “mentally handicapped children.” Additionally, some schools hesitate to enroll children who require long‐term peripherally inserted central venous catheters, central venous catheters, and intermittent leave, viewing them as abnormal and even refusing admission. These factors, often unnoticed, exacerbate the psychological pressure and caregiving burden borne by parents.

“Some people think that my child is not very smart, he does not have many friends of the same age in life, the teacher at school also suggested that he repeat the grade, I really do not know what to do.”(P12).

“The issue of schooling is really troublesome. Some schools refuse to accept us outright for fear of taking responsibility. I hope that the government can pay more attention to these children and open up green channels for them.”(P18).

## 4 Discussion

### 4.1 Enhancing Disease Education Can Encourage Parents to Respond Positively

This study suggested that although parents were worried about their children's future, most of them did not actively seek treatments for their children's cognitive impairments, which is consistent with previous studies [32]. One possible explanation is that parents have limited knowledge about cognitive impairments associated with cancer. On the one hand, parents may not be fully aware of the potential long‐term impact of cognitive impairments on their children due to limited educational backgrounds [33]. On the other hand, as national hospitals have not given sufficient attention to cognitive impairments related to childhood cancer, parents often rely on short videos and anecdotal accounts from other patients as their primary sources of information, leading to a lack of comprehensive and accurate understanding of cancer‐related cognitive impairments. Furthermore, an increasing number of parents have expressed their desire to learn more about cognitive impairments associated with childhood cancer to effectively cope with the situation [34, 35].

In recent years, an increasing number of cancer survivors and their families have expressed their desire to incorporate knowledge related to cognitive impairment into cancer treatments [34, 35]. Thus, it is advisable for medical professionals to enhance the education and dissemination of knowledge related to cancer‐related cognitive impairment. First, regular training sessions should be organized to cover topics such as the definition, etiology, and clinical manifestations of cancer‐related cognitive impairment. During these training sessions, it is crucial to avoid medical jargon and instead use simple and straightforward language to ensure effective communication, which helps enhance comprehension of the complexities of cancer‐related cognitive impairment and its impact on daily life, thereby fostering active participation and support for the patient's recovery process. Second, digital education methods like mobile push notifications, videos, and social media posts can be used to help them better understand the disease. Thirdly, for hospitalized cancer patients, methods such as posters, brochures, and personalized education sessions can be used to raise awareness about the severity of cancer‐related cognitive impairment in children, as well as the importance of regular screenings and early intervention. These efforts aim to assist parents in gaining a comprehensive understanding of cancer‐related cognitive impairment in their children and establishing positive coping strategies accordingly.

### 4.2 Strengthening Screenings and Interventions Boosts Children's Cognitive Function

Based on the results, it is suggested that parents were beleaguered by the diverse symptoms of cognitive impairment exhibited by children with cancer. Different children with cancer showed impairments in different cognitive domains, with the most significant impacts observed in areas of verbal communication, executive function, and attention. These impairments may have a significant negative impact on children's normal learning and daily life, which is similar to the findings of previous studies [36, 37, 38]. The reasons for these impairments are diverse, encompassing factors such as the type of cancer, treatment methods, and medications used for each child [39]. Furthermore, the current situation regarding the cognitive impairment of children with cancer is not optimistic, given the lack of screening and cognitive rehabilitation services specifically tailored to their needs [40]. It is suggested that early identification and targeted intervention for cognitive impairments in children with cancer can promote the recovery of their cognitive functions and enhance their long‐term quality of life [41].

Therefore, it is particularly crucial to establish a precise and highly specific system for the identification and intervention of cognitive impairment in children with cancer, who constitute a special group. Healthcare professionals can rely on hospitals to create an integrated platform for the specific risk screening and intervention of cognitive impairment in children with cancer. Following the diagnosis of cancer, this platform will conduct regular screenings and risk monitoring for cognitive impairment every quarter. For high‐risk groups such as children with brain tumors and leukemia, screenings for cognitive impairment could be conducted monthly [42, 43]. Based on the screening results, targeted intervention measures would be selected, including cognitive rehabilitation techniques such as working memory training, motor intervention, and game‐based training [44]. By shifting the focus of prevention and treatment toward cognitive impairment, we can maximize the recovery of cognitive function in these children and enhance their long‐term quality of life.

### 4.3 Multiparty Support Meets Parents' Diverse Needs

This study demonstrated that issues such as the normal enrollment of children with cognitive impairments, social isolation, and stigmatization have become concerns for parents, yet they have not received sufficient attention from society. This aligns with the findings of Yvonne and other researchers [45]. One possible explanation is the scarcity of healthcare professionals in China, which results in most medical resources being allocated to cancer treatment, with a lack of rehabilitation and support services specifically tailored for cognitive impairments related to childhood cancer [46]. Previous studies have shown that parents of affected children urgently need additional support to help their children with cognitive impairments return to school, society, and normal life [47, 48].

It is imperative for relevant departments to enhance the service system catering to children with cancer‐related cognitive impairments. First, the government must intervene promptly and adopt effective measures to allocate social resources, such as schools, in a rational manner, ensuring equal opportunities for these children to participate in school and social life. Second, hospitals, leveraging their current human resources, should facilitate the establishment of a collaborative management model encompassing hospitals, schools, and families [49, 50]. Each survivor should be assigned a designated liaison officer who serves as a key informant and communicator on cancer‐related cognitive impairments, to guide the child through a smooth transition from hospital to school and society. Lastly, society should prioritize addressing the issues of social isolation and stigmatization faced by cancer children with cognitive impairments. By harnessing the power of social media and broadcasting platforms, we can amplify public awareness of this vulnerable group and strive for their dignity, recognition, and support. Through these concerted efforts, we aspire to foster a more inclusive, nurturing, and supportive environment for these exceptional children.

#### 4.3.1 Strengths and Limitations

To our knowledge, this study may be the first qualitative study to delve into the perspectives and needs of parents regarding cancer‐related cognitive impairment in their children. Although the interview guide was composed to gain an in‐depth understanding of the challenges parents face in this regard, it also allowed for new topics to be spontaneously raised.

There are some limitations in this study. First, the small sample size may render the findings less applicable to a broader population of parents with children diagnosed with cancer. Second, the insufficient distribution of diagnosis ages is a factor, as the majority of participants were parents of children who had been diagnosed with cancer for more than 6 months. Therefore, future research should aim to balance the subjects across all time frames and expand the sample size as much as feasible.

##### Clinical Impact Statement

Parents of children with cancer‐related cognitive impairment face significant psychological burdens and they often have numerous confusions and requests regarding these symptoms. This study contributes to a deeper understanding of the multifaceted challenges and needs of children with cancer‐related cognitive impairment, laying a foundation for the development of intervention strategies that may also benefit other traumatized groups. The findings of this study provide valuable suggestions for parents, healthcare professionals, and government policymakers regarding children with cancer‐related cognitive impairment.

## Author Contributions

**Tao Liu:** investigation (equal), methodology (equal), writing – original draft (equal), writing – review and editing (equal). **LiFang Xu:** data curation (equal), formal analysis (equal), software (equal). **Yuqing Shen:** formal analysis (equal), investigation (equal), visualization (equal). **Juan Yao:** formal analysis (equal), software (equal). **Zefang Chen:** project administration (equal), validation (equal). **Lin Mo:** project administration (equal), writing – review and editing (equal).

## Conflict of Interest Statement

The authors declare that they have no financial or non‐financial interest to disclose.

## Data Availability

The data that support the findings of this study are available from the corresponding author upon reasonable request.
